# Exogenous Ketones and Lactate as a Potential Therapeutic Intervention for Brain Injury and Neurodegenerative Conditions

**DOI:** 10.3389/fnhum.2022.846183

**Published:** 2022-04-28

**Authors:** Naomi Elyse Omori, Geoffrey Hubert Woo, Latt Shahril Mansor

**Affiliations:** Health Via Modern Nutrition Inc. (H.V.M.N.), San Francisco, CA, United States

**Keywords:** ketones, exogenous, lactate, traumatic, brain, injury, neurodegenerative, metabolism dysfunction

## Abstract

Metabolic dysfunction is a ubiquitous underlying feature of many neurological conditions including acute traumatic brain injuries and chronic neurodegenerative conditions. A central problem in neurological patients, in particular those with traumatic brain injuries, is an impairment in the utilization of glucose, which is the predominant metabolic substrate in a normally functioning brain. In such patients, alternative substrates including ketone bodies and lactate become important metabolic candidates for maintaining brain function. While the potential neuroprotective benefits of ketosis have been recognized for up to almost a century, the majority of work has focused on the use of ketogenic diets to induce such a state, which is inappropriate in cases of acute disease due to the prolonged periods of time (i.e., weeks to months) required for the effects of a ketogenic diet to be seen. The following review seeks to explore the neuroprotective effects of exogenous ketone and lactate preparations, which have more recently become commercially available and are able to induce a deep ketogenic response in a fraction of the time. The rapid response of exogenous preparations makes their use as a therapeutic adjunct more feasible from a clinical perspective in both acute and chronic neurological conditions. Potentially, their ability to globally moderate long-term, occult brain dysfunction may also be relevant in reducing lifetime risks of certain neurodegenerative conditions. In particular, this review explores the association between traumatic brain injury and contusion-related dementia, assessing metabolic parallels and highlighting the potential role of exogenous ketone and lactate therapies.

## Introduction

Traumatic brain injuries (TBI), defined as an acute injury to the brain tissue due to external force, are the leading trauma-related cause of death and disability worldwide. Annually, it is estimated that 69 million people will experience a TBI (Dewan et al., 2019) with the greatest burden of disease falling on low- and middle-income countries (LMICs) and the highest incidence in North America and Europe. Concerningly, TBIs are not only associated with a significant mortality rate of 30%–40% across all age groups (Maas et al., 2017) but poor functional outcomes for survivors, who are significantly more likely to live with long-term conditions secondary to the TBI like epilepsy, post-traumatic neuropsychiatric symptoms, motor and cognitive deficiencies, and an increased risk of neurodegenerative diseases later in life. As such, there is a strong impetus within the community for identifying treatment options that can help to either improve mortality rates, reduce the impact of short- and medium-term functional impairment in survivors, and mitigate the risk of developing secondary diseases long-term.

It is known that post-injury, TBIs are associated with several metabolic changes and cellular perturbations, all of which are related to changes in the brain’s ability to utilize its preferred metabolic substrate: glucose. Broadly speaking, these include reduced oxygen and glucose uptake, suppressed glucose metabolism, and hence decreases in adenosine triphosphate (ATP) production. More specifically, they include increased utilization of the pentose phosphate pathway, activation of poly-ADP ribose polymerase, and inhibition of a key glycolytic enzyme glyceraldehyde dehydrogenase. Under these conditions, glucose becomes less energetically favorable as a metabolic substrate, and the balance instead shifts in favor of alternative energy sources that are not typically utilized by the brain including ketones, lactate, and acetyl-L-carnitine (Scafidi et al., 2010; Ferreira and McKenna, 2017). Mitochondrial dysfunction associated with reduced ATP synthesis rates has also been observed *via* altered mitochondrial dehydrogenase activity (Pandya et al., 2019). Concentration and activity of mitochondrial enzyme pyruvate dehydrogenase (PDH) have also shown to be significantly reduced following TBI, which may contribute to reduced cerebral glucose and oxygen consumption as supported by hyperoxidation of mitochondrial electron transport components and nicotinamide adenine dinucleotide observed during reperfusion (Martin et al., 2005; Sharma et al., 2009).

Ketones and lactate are of particular interest because they have been shown to be able to replace glucose as the dominant metabolic substrate for brain energy metabolism (Patet et al., 2016; Taher et al., 2016). Promisingly, recent developments in nutritional composition and formulation enable their acute and rapid exogenous delivery. The effects of ketones have been traditionally studied *via* the administration of a ketogenic diet (KD) to promote endogenous ketone production (Wheless, 2008). However, recent advances in physiology and sports science have led to the commercial availability of research-grade exogenous ketone preparations that can reliably elicit a strong ketogenic response comparable to that of KDs in only a fraction of the time (Mansor and Woo, 2021). This review seeks to understand the potential of glucose alternates, particularly exogenous ketone and exogenous lactate, as therapeutics for TBIs. While it is acknowledged that the primary pathophysiology of acute TBIs and progressive neurodegenerative conditions fundamentally differs, the review also seeks to highlight some of the secondary metabolic commonalities between TBIs and the neurodegenerative conditions they are often associated with in later life such as Alzheimer’s disease. A link is proposed, whereby metabolic modulation and neuroprotective effects conferred by ketone bodies in TBIs might be considered as an avenue for future research in both potentially improving functional outcomes in those with a known neurodegenerative condition or exogenously administering ketone bodies in high- risk groups as a prophylactic against future neurodegenerative disease. The current understanding of cerebral metabolism in the healthy brain vs. one afflicted with a TBI is first presented, followed by a discussion of the different ways in which ketosis and elevated lactate can be induced and an assessment of these exogenous methods in relation to TBIs. Owing to the relative difficulty of studying TBIs, due consideration is paid to promising animal models as well as human studies.

### Background on Ketones/Cerebral Metabolism of Ketones

Cerebral metabolism of KBs has been studied previously in preclinical work and in the context of neonates for whom KB metabolism is upregulated and KBs are preferentially utilized as precursors for lipid and amino acid synthesis (Edmond et al., 1985; Cotter et al., 2011). Unlike fatty acids, which also cross the blood-brain barrier, KBs can and are metabolized by the brain as a source of energy during periods of glucose shortage. They are also used in the brain as substrates for the synthesis of molecules like lipids *via* acetoacetyl-CoA synthetase or amino acids.

Cerebral KB metabolism is regulated by three main factors: the global blood concentration of KBs, which is considered the most significant factor; the blood-brain barrier; and the concentration and activity of KB-metabolizing enzymes (Morris, 2005). In non-fasted humans, circulating KB concentrations are typically low, which is the primary reason for needing to fast, follow a ketogenic diet, or administer exogenous ketones to increase blood KB concentrations. Clinical studies have demonstrated a linear correlation between the arterial concentration of KBs and cerebral KB uptake (Mikkelsen et al., 2015). Circulating KBs are then transported into the brain *via* the BBB. The BBB features a transporter for short-chain monocarboxylic acids (Gjedde and Crone, 1975). Different KBs are transported through the BBB at different rates with AcAc uptake being twice as fast as 3-hydroxybutyrate (Hawkins et al., 1986). Preclinical studies have indicated an age dependency on BBB permeability to KBs with suckling rates having higher permeability than adults, but a tendency for increased permeability is demonstrated following periods of extended fasting or use of a high-fat diet. This is thought to be linked to the expression of monocarboxylic acid transporters (MCT), with MCT1 expression being up to 25-times higher in suckling rats than in adults (Gerhart et al., 1997; Pellerin et al., 1998a; Leino et al., 1999) and increased eight-fold in adult rats following dietary intervention (Leino et al., 2001). Finally, within the brain, there are various mitochondrial enzymes utilizing KBs including succinyl-CoA 3-oxoacid, CoA transferase, and mitochondrial acetoacetyl-CoA thiolase, although they are not considered the main factor regulating KB metabolism in the brain. In preclinical studies, enzyme activity is seen to be low at birth, rising through the suckling period, reaching a maximum at weaning, and then falling to lower levels in adulthood.

Preclinical studies indicate that site-specific cerebral KB uptake may vary with age. KB uptake appears to be more homogenous in neonates than adults (Nehlig et al., 1991), where autoradiography studies have demonstrated preferential uptake in regions of higher vasculature and metabolic rate like the deep cerebral cortex, pituitary and pineal glands, and colliculi (Hawkins et al., 1986). Randomized control studies using PET have demonstrated that under constant infusion of 3-hydroxybutyrate, cerebral glucose utilization and uptake are reduced by 14% but oxygen consumption remains constant, and cerebral blood flow is increased by 30%, indicating a potential mechanism for the neuroprotective effect of KBs due to increased blood supply (Svart et al., 2018).

Beyond their role as metabolic alternatives, KBs are increasingly understood to be capable of regulating neuronal excitability and gene expression (García-Rodríguez and Giménez-Cassina, 2021). These additional effects include the regulation of neuronal firing rates *via* increased ATP: ADP ratios (Devivo et al., 1978), upregulated expression of molecules such as brain-derived neurotrophic factor (BDNF; Sleiman and Chao, 2015) and mitochondrial glutathione (Jarrett et al., 2008), and the potential ability to modulate cell signaling pathways (Zou et al., 2016; Katsu-Jiménez and Giménez-Cassina, 2019).

## Cerebral Metabolism During TBI

### Glucose and the Healthy Brain

Functional brain tissue is comprised of billions of neurons and glia (von Bartheld et al., 2016). Neurons are polarized cells that can communicate with each other through the conduction of nerve impulses, while glial, which include astrocytes, microglia, and oligodendrocytes, perform various supportive functions (Jäkel and Dimou, 2017). Healthy brain function is dependent on having the correct diversity, balance, and density of neuronal populations in different regions of the brain. Imbalances on this front have been noted as a common feature of various neuropsychiatric conditions (Ramocki and Zoghbi, 2008).

In a normally functioning brain, it is traditionally understood that glucose is the primary substrate for energy metabolism. Despite accounting for around 2% of total body weight, the brain demands a disproportionately high amount of glucose and oxygen relative to its size (Rolfe and Brown, 1997), utilizing around 20% of available glucose-derived energy, which makes it the largest consumer of glucose in the body. Clinically, the importance of glucose in brain function has been demonstrated in studies during the 1970s of patients experiencing insulin-induced hypoglycemia, where the administration of exogenous glucose was found to dramatically reverse the progressively worsening adrenergic symptoms and neuroglycopenic signs of hypoglycemia (Sokoloff, 1973), as summarized in Table 1, which in the worst case can lead to loss of consciousness (Kalra et al., 2013). Conversely, other substances like glycerol, ethanol, lactate, acetate, β-hydroxybutyrate, and galactose were shown to have no effect (Sokoloff, 1973).

**Table 1:** Symptoms arising from depleted arterial plasma glucose levels (Mergenthaler et al., 2013).

Arterial plasma glucose levels (mmol/L)	Symptoms
4#x02013;6	Normal
3	Endocrine responses, symptoms of hypoglycemia (incl. anxiety, palpitations, hunger, tremor, sweaty, dizziness, and weakness)
2.6	Cognitive dysfunction, difficulty speaking, blurred vision
1.7	Mild confusion, delirium
1.1	Cognitive failure, stupor, seizures
#x0003C;0.6	Coma, death

**Figure 1:**
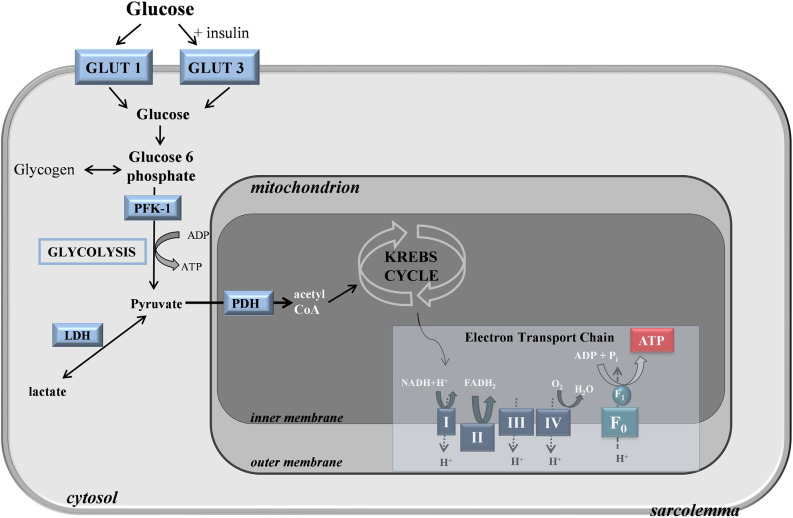
Glucose transport and metabolism in the cell.

The brain itself has a low capacity for glucose storage in the form of glycogen, so glucose uptake predominantly occurs through the selectively permeable blood-brain barrier (BBB; Gruetter, 2003). As the BBB stringently restricts the entry of neuroactive compounds like glutamate or glycine, such molecules must be synthesized inside the brain, in part justifying the brain’s high demand for glucose (Patching, 2017). Within the brain, an extensive network of glucose transporters mediates the uptake of glucose across endothelial membranes into extracellular fluid *via* GLUT1 transporters. GLUT1 transporters also mediate glucose uptake from the extracellular fluid into astrocytes, which after neurons have the highest demand for glucose, microglia, and oligodendrocytes, while GLUT3 transporters meditate rapid uptake of glucose into neurons (Figure 1). Modeling studies indicate that the majority of glucose diffuses from endothelial cells through gaps in astrocytic dendrites and into the extracellular fluid, from where it is able to diffuse globally throughout the brain for uptake by GLUT3 transporters into neurons (Simpson et al., 2007). Glucose also enters astrocytes through their dendritic feet where it diffuses into other coupled astrocytes.

Upon entering the cytoplasm, glucose is metabolized through a process termed glycolysis, or the Embden-Meyerhof pathway, to form pyruvate, adenosine triphosphate (ATP), and nicotinamide adenine dinucleotide (NADH; Chaudhry and Varacallo, 2021). Glycolysis is a complex 10-step process consisting of non-reversible and reversible steps that are closely interrelated with other metabolic cycles like the Kreb’s cycle and the pentose phosphate shunt pathway (PPP) due to the multipurpose intermediate compounds formed. The intermediate steps of glycolysis can be split into the preparatory or investment phase and the yield phase. In the preparatory phase, 2 moles of ATP are consumed during the enzymatic conversion of intracellular glucose by hexokinase to α-d-Glucose 6-phosphate (G6P) and the phospho fructokinase 1 (PFK-1) catalyzed conversion of fructose 6-phosphate (F6P) to β-d-fructose 1,6-bisphosphate (F1,6BP). During the yield phase, 4 ATP molecules per molecule of glucose are produced, resulting in a net production of 2 ATP per 1 glucose molecule. As well as being a substrate for the PPP, which generates nicotinamide adenine dinucleotide phosphate (NADPH), G6P is utilized in astrocytes where it acts as a precursor for glycogen, which is a polymeric form of glucose that functions as a short-term energy reserve. Rates of glucose metabolism vary locally depending on the functional requirements of different regions.

Historically, this understanding, established as early as the 1930s, has been informed by *in vivo* human (Gibbs et al., 1942; Kety and Schmidt, 1948) and animal studies (Himwich and Nahum, 1932) of healthy subjects measuring arteriovenous (AV) differences or gradients across the brain and applying the Fick principle to calculate cerebral oxygen consumption. AV ratios of the reactants and products of the glucose oxidation reaction (C_6_H_12_O_6_ + 6 O_2_ → 6 CO_2_ + 6 H_2_O) can be used to stoichiometrically infer substrate utilization, with a respiratory quotient (RQ) [(AV)_CO_2__/(AV)_O_2__] of 1 and an oxygen-glucose index (OGI) or metabolic ratio (MR) [(AV)_O_2__/(AV)_glc_] of 6 denoting total glucose oxidation. An MR over 6 implies the aerobic metabolism of alternative substrates, while less than 6 implies the anaerobic metabolism of glucose. In healthy patients, the MR has been shown to be around 6 (Glenn et al., 2015).

### Mechanisms of Damage

The brain is responsible for the most functionally complex and energy-demanding tasks in the body. As alluded to previously, optimal performance is dependent upon a delicate balance of cell populations, metabolic substrate availability, and neuronal interconnectedness. This is achieved, in part, by chemically demarcating the brain from the rest of the body with the BBB, creating an insulated environment within which cerebral homeostasis can be more readily maintained (Daneman and Prat, 2015). A complicating matter is the brain’s limited capacity for glycogen storage and its need for a reliable glucose supply across the BBB, which is compounded by the fact that its pathways for generating energy are fundamentally linked to and therefore affect neurotransmitter function. This makes the protective feature of the BBB something of a double-edged sword when the brain experiences any physical insult (Alves, 2014).

Traumatic brain injury can be defined as an acute injury to the brain tissue due to external force. Depending on the severity of the injury, TBI may cause acute damage to cellular membranes, raised intracranial pressure from skull fractures or bleeding, disruption to the BBB, dysfunctional release of neurotransmitters, and disruption to intercell gradients necessary for neuronal signaling (Toth et al., 2016). Given the brain’s sensitivity to its local environment, even very minor TBIs can be associated with metabolic changes and cellular perturbations that can develop over time into other forms of neurological malfunction. In summary, metabolic dysfunction in TBI patients can be attributed to: (1) structural perturbations leading to ischemia; and (2) alterations to neuronal circuits.

While the prevalence of severe ischemia in patients receiving treatment in modern neuro-intensive care units (NICU) is relatively low, structural perturbation of brain tissue means there is an inevitably elevated risk of microvascular ischemia, raised intracranial pressure, and compromised vascular autoregulation, which in combination can still lead to hypoxia and incomplete perfusion of the brain in apparently hemodynamically stable patients (Toth et al., 2016; Khellaf et al., 2019). TBI is also associated with a change in neuronal circuits occurring over both the short and long term. Key factors contributing to this include changes in the population and relative concentration of neurotransmitters and receptors and injury-specific depletion of certain cell types (Bramlett and Dietrich, 2015).

### Impaired Glucose Handling Post-TBI

From a metabolic perspective, characterizing the full nature and extent of changes experienced during TBIs can be challenging due to the intrinsic limitations of methods capable of measuring glucose metabolism *in vivo*. These include practical factors like their invasiveness, use of radiotracers, and duration of data acquisition, which may be unfeasible to perform in patient populations who are more likely to be receiving complex medical treatment, and technical limitations like spatiotemporal resolution and lack of glucose specificity. Studies in both human and animal populations indicate that TBIs are associated with altered uptake and metabolism of glucose and oxygen. The term “altered uptake” is selected deliberately instead of summarizing any global trends, as a major caveat of any changes reported in observational studies is that they are thought to be dependent on the individual’s metabolic state prior to the incident and the nature of their specific injury (Greco et al., 2020).

In the period immediately preceding injury, cerebral blood flow is understandably decreased, reducing the potential for oxygen utilization (Kawai et al., 2008). The risks of hypoxia are already well-understood and acute mitigation of them is already established as a priority in the emergency treatment of TBIs (Godoy et al., 2018; Kramer et al., 2018). Of greater contention is the utilization of glucose post-TBI. There is a body of studies that have identified an increased demand for glucose, which is accompanied by an increase in the proportion of glucose that is anaerobically metabolized as opposed to aerobically (Holbein et al., 2009), as is the case in healthy controls. This could be due to either an acute need for glucose-derived energy to compensate for the early-stages of injury or an increased demand from reparative metabolic cycles like the PPP. Increased glucose uptake has been observed both globally and focally, especially where the TBI is associated with a focal mass lesion. Increased glucose uptake also appears to be more pronounced in severe TBIs (Bergsneider et al., 2001).

Equally, there are studies that have observed reduced glucose uptake. AV studies by Cruz (1995) and Glenn et al. (2003) showed reduced glucose uptake. Glenn et al. (2003) also observed a depression in oxygen availability and utilization resulting in an MR of less than 6, implying greater anaerobic metabolism of glucose. Microdialysis studies, which involve continuous *in vivo* sampling of extracellular fluid from the brain *via* catheters, Vespa et al. (2003) observed a trend of declining mean glucose concentrations over a 5-day period post-injury in 30 patients with severe TBIs and Yokobori et al. (2020) found a trend of decreasing glucose until the 4th day post-injury after which it appeared to increase. Neuroimaging studies have provided greater insight. In quantitative FDG-PET and H_2_^15^O-PET studies, Wu et al. (2004) were able to localize significantly decreased glucose uptake in the cortical gray matter but not white matter, along with decreased gray matter hexokinase activity, indicating that seemingly contradicting observations regarding glucose uptake in global sensing methods could perhaps be attributed to spatiotemporal variations. Across different studies, glucose uptake has been used as a predictor of mortality and outcome. Vespa et al. (2003) associated lower extracellular glucose and higher extracellular glutamate with worse outcomes 6 months post-TBI. A large prospective study following 223 patients by Timofeev et al. (2011) was also able to show that elevated glucose, lactate: pyruvate ratios, and intracranial pressure were all positive independent predictors of mortality, while elevated pyruvate was a negative predictor of mortality.

It is uncertain to what extent altered glucose and oxygen uptake impacts rates of glycolysis. Studies showing increased levels of the products of anaerobic and aerobic glycolysis (i.e., lactate and pyruvate respectively) imply hyperglycolysis by both pathways (Neppl et al., 2001; Buitrago Blanco et al., 2016). Conversely, ^13^C-labeled studies indicate glucose is preferentially funneled into the PPP (Dusick et al., 2007; Jalloh et al., 2015), which would contradict assumptions that increased glucose uptake correlates directly with hyperglycolysis. In either case, long-term there appears to be a trend of hypometabolism months to years following the TBI. Studies 6-months post-TBI have demonstrated decreased glucose uptake in the bilateral frontotemporal lobes, thalamus, and right cerebellum, which in a study of IQ by Kato et al. (2007) was also associated with lower IQ scores demonstrating the broader negative functional impact on cognitive processing. Follow-up studies over a year after TBI involving diffuse axon injury showed decreased cerebral oxygen uptake in a majority of patients (Shiga et al., 2006).

Ultimately, hyper- and hypoglycemia can both aggravate brain damage in TBI patients, with hyperglycemia seemingly inducing tissue acidosis, immunosuppression, and oxidative stress, and hypoglycemia impairing metabolic function through reduced energy supply as well as cortical spreading depolarizations. Avoiding both in favor of normoglycemia, which is thought to be around 6–9 mM arterial and 1–5 mM brain glucose (Meierhans et al., 2010), is associated with significantly better clinical outcomes in severe TBI patients but can be practically very challenging and over- or underfeeding is common. For this reason, there has been interest in nutritionally supporting TBI patients with sources other than glucose to reduce the risk of clinically-induced or propagated hypo- or hyperglycemia. Two options that have surfaced are lactate and ketones.

### Increased Involvement of Lactate

At a certain point, global levels of glucose within the brain appear to deplete and lactate begins to emerge as an important metabolic substrate. Elevated lactate in the brain following TBI is recognized and associated with overall poorer clinical outcomes (Carpenter et al., 2015). Within the brain, it is thought that lactate is regulated by the astrocyte-neuron lactate shuttle (ANLS), which posits that lactate is produced within the BBB and shuttled dynamically between glia and neurons (Pellerin et al., 1998b). During neuronal activity, glutamate is expelled into the synaptic cleft, which, it is then hypothesized, triggers glucose uptake when taken up by astrocytes leading to the production of lactate that is transported *via* monocarboxylate transporters for neurons to utilize (Tarczyluk et al., 2013). Although the importance of lactate utilization by cerebral neurons *via* the astrocyte-neuron lactate shuttle hypothesis in healthy humans has previously been a topic of debate (Chih et al., 2001), more recent studies focusing on the modulation of the ANLS have demonstrated its potential neuroprotective role in neurodegenerative diseases (Liu et al., 2017; Sun et al., 2020). However, Brooks and Martin (2015) also demonstrated a massive mobilization of lactate from corporeal glycogen reserves in TBI patients during periods 5–7 days post-TBI where oxygen and glucose uptake are decreased in a series of comprehensive [6,6-2H2]glucose, [3–13C] lactate, and AV studies. Compared to healthy controls who exhibited a rate of 15.2% ± 2.8% for gluconeogenesis (GNG) from lactate, GNG from lactate in TBI patients accounted for 67.1% ± 6.9%, indicating a huge temporary dependence on lactate. This could be interpreted to mean that beyond maintaining normoglycemia, patients with TBIs may require additional dietary support in the form of lactate supplementation to optimally promote recovery.

Lactate is an important metabolic intermediate produced during glycolysis and utilized as the primary substrate in mitochondrial respiration. Similar to KBs, lactate is transported into the brain by monocarboxylate transporters (Bergersen, 2015) at a rate that, in healthy humans, has been shown to increase as a function of plasma lactate concentration (Ide et al., 1999, 2000). KBs appear to be preferred over lactate as a cerebral metabolic substrate as evidenced by accumulations of lactate following a ketogenic diet where KBs become more readily available (Pan et al., 2000). In the brain, it binds to the lactate receptor or hydroxycarboxlic acid receptor (HCAR1) and regulates levels of cAMP (Bergersen, 2015). Neurons and astrocytes both contain lactate transporters that enable cellular uptake of lactate, and neuronal mitochondria feature components of the mitochondrial lactate oxidation complex (mLOC) required for its oxidation. It is often overlooked as a metabolic substrate because the body tends to remove it at a comparable rate to which it is produced (Dienel, 2012). Sensing modalities with low dynamic ranges are unable to capture the body’s rapid lactate turnover rate, and lactate concentration across longer time periods (e.g., a day) betray little information regarding its production and utilization within the body.

### Ketones as a Therapeutic Alternative Metabolic Substrate Post-TBI

In certain situations, the brain has also been found to be capable of fulfilling metabolic requirements by exploiting ketones. A ketone is a functional group containing a carbonyl structure (R_2_C=O, where R is an associated hydrocarbon group). Preclinical studies in rats on a diet of ketone-supplemented glucose have been shown to recover metabolically faster following TBI (Hawkins et al., 1971; Cotter et al., 2011), hinting at the important role of ketones in providing energy as an important alternative substrate than glucose.

The use of ketones is theoretically appealing as it offers a short-term option for the brain to bypass glucose-derived energy metabolism pathways that become fundamentally disrupted post-TBI, as well as triggering a cascade of protective features that could improve long-term functional outcomes in patients. Ketones are relatively readily incorporated into the TCA cycle and can increase the rate of ATP hydrolysis while decreasing the rate of free radical production in the cytosol and mitochondria (Cotter et al., 2013). Unlike lactate, which is produced continuously as a result of glycolysis and hence can be drawn from significant stores throughout the body in the event of a TBI, studies of ketone bodies require subjects being either placed in a ketogenic state *via* the use of dietary interventions or exogenous supplementation (Nasser et al., 2020).

Two particular ketone bodies (KBs) of interest that play an important role in ketogenesis are β-hydroxybutyrate (βHB) and acetoacetate (AcAc). Ketogenesis is catalyzed by 3-hydroxymethylglutaryl-CoA synthase (HMGCS2), a mitochondrial matrix enzyme that generates HMG-CoA from β-oxidation-derived acetoacetyl-CoA and acetyl-CoA. Following the lyase-dependent release of acetyl-CoA, AcAc is formed. AcAc is later reduced to βHB by another mitochondrial matrix enzyme D-βOHB dehydrogenase (BDH1). βHB and AcAc are capable of undergoing interconversion, with βHB oxidizing back to AcAc by BDH1 through mass action. The ratio of circulating βHB and AcAc is regulated largely by the liver and is proportional to the ratio of reduced and oxidized nicotinamide adenine dinucleotide (NADH/NAD^+^; Stagg et al., 2021). Ketosis is then defined as a metabolic state where the concentration of blood βHB is ≥ 0.5 mM.

Although glucose is the dominant cerebral metabolic substrate, there are a number of studies that show KBs become a preferential fuel source during periods of low glucose availability (i.e., starvation) when the body is instead in a state of ketosis. Experimental studies show that uptake of KBs into the brain is largely dependent on systemic blood concentration (Balasse and Féry, 1989; Hasselbalch et al., 1995; Mikkelsen et al., 2015); in other words when in a state of systemic ketosis, the rate of KBs uptake to the brain increases, as confirmed by neuroimaging PET studies in rats showing that the uptake of AcAc doubles during periods of ketosis (Bentourkia et al., 2009; Pifferi et al., 2011). This may also be achieved by endogenous or exogenous ketone supplementation, with studies of cerebrospinal fluid (CSF) showing that concentrations of βHB increase to become comparable to that of a 24 and 40 h fast after dosing with an exogenous preparation of 4 and 8 g of KBs respectively (Plecko et al., 2002). The mechanism by which this happens is not entirely clear, but some rodent studies indicate that KBs enter the brain through monocarboxylate transporters (Nehlig, 2004).

## Inducing Ketosis

It is possible to elevate ketone bodies both endogenously and exogenously. Such therapeutic strategies have been authoritatively reviewed by Camberos-Luna and Massieu (2020), but can be summarized into three main categories, which are namely the use of a ketogenic diet (KD), feeding augmentation (including calorie restriction and intermittent fasting), and exogenous supplementation of ketone bodies, KB derivatives, and medium chain triglycerides (MCTs). Generally, endogenously induced ketosis requires the use of extended treatments before ketogenesis is observed, while the use of exogenous supplementation can increase plasma ketones in a matter of hours but require ongoing dosing to maintain the effect (Cox et al., 2016; Stubbs et al., 2017; Evans et al., 2018; Koppel and Swerdlow, 2018).

### Endogenous Methods

The KD has been recognized as a treatment modality as far back as the 1920s when it was used to regulate seizures in children with epilepsy (Höhn et al., 2019) during a time when pharmacotherapies were not yet widely available. Since then, multicenter studies have demonstrated the efficacy of the KD in reducing not only seizures (Vining et al., 1998) but also conferring cognitive improvements (van Berkel et al., 2018), and a neuroprotective effect on cellular damage (Gasior et al., 2006). While there is some variability in the exact composition of a KD, it is generally accepted that it is comprised of low carbohydrates (i.e., 5%–19%; Gasior et al., 2006; van der Louw et al., 2017) along with a combination of high fat (i.e., 71%–90%) and low to moderate protein intake [Institute of Medicine (US) Committee on Nutrition]. For example, the Johns Hopkins KD protocol specifies a 4:1 ratio of fat to protein plus carbohydrates (Wheless, 1995). The time required to see elevated KB varies depending on the model. In rodents, an effect may be seen within 7 days (Prins et al., 2005; Modica et al., 2021) depending on whether a controlled brain injury has been introduced or not. In human studies, elevated KBs may be also achieved in as little as a week (Pinckaers et al., 2017) or within 24–48 h if fasting is employed, although it is worth noting that this is highly dependent on the diet formulation and patient compliance, and in practical terms may rather be seen after a period of months depending on the subject’s age and previous medical history (Gilbert et al., 2000; van Delft et al., 2010).

A similar effect can also be observed *via* augmenting feeding patterns either by intermittent fasting (IF), caloric restriction (CR), or a combination of the two. Similar to the KD, a rudimentary appreciation of their potential therapeutic value has existed since the early 20th century (Lennox and Cobb, 1928; Cahill et al., 1966; Owen et al., 1967; Sokoloff, 1973; Appleton and DeVivo, 1974). Various protocol exists for intermittent fasting including fasting on alternate days, restricting food intake to a 6–8 h window on a daily basis, and the 5:2 method of fasting on two consecutive days each week (Camberos-Luna and Massieu, 2020). Dependent on the protocol, both IF and CR have been shown to increase KBs. In rodent studies, they have also been associated with a range of desirable health outcomes including increased lifespans, weight loss, improved glucose tolerance, and increased lean mass (Anson et al., 2003; Longo and Mattson, 2014; Singh et al., 2015; Gotthardt et al., 2016).

### Exogenous Methods

Exogenous means of inducing ketogenesis involve supplementation with a preparation of ingestible exogenous ketone (EK) substances. Exogenous supplementation does not necessarily require further dietary modification and represents a controlled and reproducible manner of inducing ketosis. A range of different types of natural and synthetic EKs exist, which utilize different ketone formulations.

#### Ketone Salts

Although it would initially seem that directly administering KBs of interest (i.e., R-βHB and AcAc) would be the most intuitive form of exogenous supplementation, it happens these molecules in their free acid form are unstable and unsuitable for inducing sustained states of ketosis. A simple option for reformulation is the use of their salt forms by buffering the ketone acids with Na^+^, K^+^, or Ca^2+^. Evidence regarding the efficacy of salt formulations is variable. In healthy candidates, ketone salt formulations with caffeine and amino acid additives produced comparable high-intensity exercise performance to keto-adapted athletes (Kackley et al., 2020). Studies in similar healthy participants also found that ketone salt formulations did not have a negative effect on ketogenic diets or sporting performance and were associated with a perceived benefit (Prins et al., 2020; Buga et al., 2021). Equally, studies demonstrating limited utility with ketone salt supplementation showed only modest increases in plasma *R*-βHB and no improved cognitive response (Waldman et al., 2018) or enhanced physical performance under stress (O’Malley et al., 2017; Evans et al., 2018; Waldman et al., 2020). Additionally, elevated Na^+^ (or similar) intake from large, sustained supplementation with ketone salts can lead to sodium imbalances and gastric hyperosmolarity.

#### MCTs

An alternative class of substances is medium chain triglycerides (MCTs), which are KB precursors made up of triglycerides with two to three fatty acids and an aliphatic tail of 6 to 12 carbon atoms. MCTs are a readily available source of high energy. They are often discussed in relation to the closely related structure long-chain triglycerides (LCTs), another widely available type of dietary lipid that is absorbed and metabolized differently from MCTs. Most notably, MCTs can be metabolized into KBs in a way that LCTs cannot. LCTs are dependent on the fatty acid β-oxidation pathway and are usually hydrolyzed in the gut to form long-chain fatty acids (LCFAs) that re-esterify again into triglycerides and are then transported to the liver *via* the lymph system. In contrast, MCTs are predominantly absorbed as medium-chain fatty acids (MCFAs) directly into the blood where they undergo first-pass hepatic metabolism *via* the portal vein to form acetyl CoA and KBs. When given as a single energy source (i.e., in the absence of carbohydrates) to healthy human subjects, isotope tracer studies show that around 40% of MCTs are oxidized and becomes 62% when administered in conjunction with carbohydrates and protein (Knottnerus et al., 2021). A side-effect of overconsumption of MCTs is gastrointestinal (GI) discomfort (Thorburn et al., 2006), which can be addressed by a gradual increase of MCT supplements over an extended period of weeks.

There are indications that combining ketone salts and MCTs could be potentially useful. In a rodent study, Kesl et al. (2016) tested the effects of a 28-day course of an exogenous ketone supplement made up of an Na/K β-hydroxybutyrate (βHB) mineral salt (BMS) in a 1:1 ratio with an MCT oil comprised of approximately 65% caprylic triglyceride and 45% capric triglyceride. When compared with an MCT monotherapy, the combined BMS+MCT supplement was shown to elevate βHB to a similar extent to MCT without causing the GI side-effects observed in the MCT cohort. It is thought that this could be due to the combination of KBs being delivered directly *via* the salts as well as the sustained ketogenesis being stimulated by the MCT.

#### Ketone Esters and Derivatives

There are more EK substances that are being investigated and developed for safety and efficacy for human consumption. These include 1,3-butanediol, ketone monoesters, acetoacetate diesters, and bis-hexanoyl-butanediol diester. 1,3-butanediol is an organic diol that was previously approved by the Food and Drug Administration (FDA) as a solvent for food flavoring agents and recently obtained the Generally Recognized as Safe (GRAS) for human consumption (America, 2020). Notably, it can be metabolized in the liver to form βHB and racemic 1,3-butanediol has been shown to produce similar ketotic effects to MCTs and ketone salts in rats (D’Agostino et al., 2013; Kesl et al., 2016) while being apparently tolerable (Scala and Paynter, 1967). It can also be used as a backbone component of ketone esters. Ketone esters are comprised of KBs bonded by ester bonds to a backbone molecule to form a substance with a stable shelf life but that can be broken down by gastric esterases to generate KBs in their free acid form. While the other diesters are still very early in their development and commercialization stages, there is some data to suggest that they are tolerable and potentially efficacious for specific use cases such as cancer cachexia (Koutnik et al., 2020; Chen et al., 2021). These different exogenous ketone supplements are capable of reliably producing a strong dose-dependent increase in KBs.

A final consideration in the production of exogenous supplements is the implications of enantiomers. βHB exists in two chiral forms (S- and R-βHB) but is most commonly sold as a racemic mixture, given that such mixtures are generally cheaper to produce than enantiopure forms. R-βHB is produced endogenously in the liver when the body is being restricted of carbohydrates, while S-βHB is only organically present in exceedingly small amounts as a minor intermediate during β-oxidation (Haces et al., 2008). The precise role and effect of the two enantiomers is still a topic of discussion. However, it has been noted that the metabolic fate of the two enantiomers appears to be different with S-βHB being in circulation longer than R-βHB, suggesting that S-βHB is perhaps less efficient as a metabolic fuel due to lower oxidative rates (Webber and Edmond, 1977; Stubbs et al., 2017), but also potentially playing a greater role in redox signaling due to its longer circulation times. This could be a consideration when formulating EK preparations.

## Potential of Neuroketotherapeutics for TBIs

Neuroketotherapeutics refers to the use of ketosis in treating neurological conditions. Preclinical meta-analyses of mammalian studies have pointed toward the potent global neuroprotective effect imparted by ketosis as measured by mortality, neuronal dysfunction, and damage (Gambardella et al., 2021). The following section reviews recent advances in the field of neuroketo therapeutics for TBIs focusing on the KD as well as exogenous formulations.

### Ketogenic Diet

The efficacy of the KD as a treatment for TBI in animal studies has been reviewed by McDougall et al. (2018). The nine studies selected focused on rat models with moderate to severe TBIs simulated with controlled cortical impact. KD appears to have a positive effect on apoptosis and cognitive function. Apoptosis is comparatively difficult to study in human populations as it often utilizes post-mortem brain slices to estimate neuronal populations, but it can be readily studied in rat populations. Markers of apoptosis were found to be decreased in post-mortem cortical and the hippocampal stains of TBI rats fed a KD vs. a Standard Diet (SD) in studies using various different markers including bax-mRNA and bax protein levels, cytochrome c mitochondrial release, TUNNEL-positive cells, and caspase-3 expression (Gillardon et al., 1999; Ott et al., 2002; Prins et al., 2005). A study by Schwartzkroin et al. (2010) focusing on hippocampal slices only did not find significant differences in neuronal populations between KD- and SD-fed rats, highlighting the need for further studies. However, the broad range of markers used to demonstrate similar outcomes for apoptosis provide a compelling argument for its protective effect.

Cognitive improvements were demonstrated by Davis et al. (2008), Appelberg et al. (2009), and Har-Even et al. (2021) who used the Morris water maze (MWM) and beam walking as measures of cognitive and motor function. In both cases, rats fed the KD performed better than the SD rats at 3, 4, and 10 days post-injury. There was also weak evidence to suggest that rats on the KD had reduced cerebral water content compared to SD rats, which could be extended to indicate that the KD may reduce the effect of cerebral edema (Hu et al., 2009), which is a secondary complication of TBIs.

An interesting age-dependency was also noted in rat studies where it was suggested that the time it takes for the brain to transition from a glucose-dominant to a ketone-dominant metabolic pathway may be faster in younger populations. Appelberg et al. (2009), who compared rats receiving Controlled Cortical Impact (CCI) at postnatal day (PND) 35 and PND 75 and then fed a KD for 7-days found in motor and cognitive testing that enhanced performance was only seen in the PND 35 cohort. The finding has been corroborated by studies by Prins and Hovda (2009) and Deng-Bryant et al. (2011). Prins et al. (2005) additionally found in their study of CCI in PND 17, 35, 45, and 65 rats that ketogenic neuroprotective effects were only seen in PND 35 and 45 rats, indicating that overly young populations may also be unlikely to benefit from the dietary intervention.

To address this discrepancy, a novel KD formulation was trialed and evaluated by Thau-Zuchman et al. (2021). A diet with a fat: (carbohydrate + protein) ratio of 2:1 comprised of MCTs, DHA, low GI carbohydrates, fibers, and leucine was fed to PND 70–84 mice with a CCI for 70 days. Ketosis was confirmed through elevated β-HB plasma levels. Compared to a control group, the novel KD-supplemented cohort showed sensorimotor improvement as soon as 3 days post-TBI, improved spatial memory based on MWM performance, decrease in oxidative stress markers, decreased microglia activation, protection of oligodendrocytes, and interestingly a modulating effect on the mTOR signaling pathway. The study provides compelling evidence of how alternative KD preparations can be used to target different groups. This study in particular also benefits from a very comprehensive experimental approach that addresses cognitive and behavioral effects, metabolic effects, and pathological changes to holistically characterize their intervention. This is in comparison to many earlier studies that only manage to address one or two of these aspects at a time. Moving forward, more comprehensive studies like this will be useful in determining true effect sizes in TBI populations.

There is comparatively limited evidence supporting the benefits of the KD in humans. An early study by Robertson et al. (1991) looked at post-TBI alimentation in comatose patients with a TBI and found that patients not receiving additional glucose in a state of ketosis were utilizing KBs in 16% of their total energy consumption and had reduced evidence of glycolysis (i.e., reduced spinal fluid lactate production). Similarly, Ritter et al. (1996) was able to safely demonstrate that a KD provided sufficient calories and avoided hyperglycemia post-injury. In both cases, the studies suffer from small sample sizes and a lack of cerebral metabolic measures, meaning that the efficacy of KD as a therapy in TBIs in humans is yet to be established. However, based on animal studies, it would appear that there is potential for the KD to be used as a therapeutic intervention, with a potentially enhanced effect in younger populations. Currently, Phase I trials of the KD in adult populations have demonstrated its feasibility and safety (Arora et al., 2022), and it is likely that randomized control trials and Phase II trials in the near future will provide further insight into the applicability of KD in TBIs.

### Exogenous Ketones

The interest in the potential therapeutic application of exogenous ketone supplementation has grown in light of positive results from KD studies over the last two decades. Compared to KD, exogenous ketones could theoretically induce similar neuroprotective effects but in a shorter period of time and with less need for dietary compliance and stringency. There has been some promising data relating to the clinical efficacy of exogenous ketones in controlling related neurological conditions like seizures reportedly through activation of inhibitory ATP-sensitive channels, reduced oxidative stress, and enhanced mitochondrial function (Poff et al., 2019). For TBIs to date, there are markedly fewer studies on exogenous ketones compared to KDs, and the majority of compelling studies demonstrating their application in TBIs have been in preclinical animal studies.

Prins et al. (2004) found that rats with an introduced CCI showed an 8.5-fold increase in the cerebral uptake of βHB following a 3 h infusion when compared to both CCI rats with only endogenously available βHB and healthy controls. Accompanying this was a 10.7-fold increase in ^14^CO_2_ production and an alleviation in the TBI-induced depression of cortical ATP.

Ketone salt preparations have also been trialed. Yin et al. (2015) studied subcutaneously administered ketone salts given over a 30-min period every hour for 6-h in rats with a transient middle cerebral artery (MCA) occlusion designed to simulate an ischemic stroke. The supplementation was shown to reduce infarct and penumbral volumes in MRI scans and improve the subjects’ neurological scores in the 24 h following the injury. Tan et al. (2020) adopted a combined KD-exogenous ketone salt (KS) approach in a related model of rats with acute spinal injuries. In their study, the neuroprotective effects of the KD were not exceeded by the KD+KS combination, but in both cases neuronal and axonal cell death was minimized, and forelimb motor abilities were improved 4–6 weeks post-injury.

A topic of ongoing research in humans is the safety, efficacy, and tolerability of different preparations of EKs in TBI populations, which to date has somewhat hampered further human studies. White et al. (2020) recently showed in a small study of 20 patients with stroke, subarachnoid hemorrhage, or TBI that a ketogenic preparation enterally administered over a 6 day period was well tolerated and did not negatively impact intracranial and cerebral perfusion pressures, while still inducing ketogenesis.

Overall, a consideration for the use of exogenous ketones ties into the overall efficacy of different ketone preparations in eliciting a sustained ketogenic response. Ketone salts alone, for example, have variable efficacy in studies focusing only on their capacity for inducing ketosis, so it is perhaps questionable to what degree they might truly have an effect in TBI populations. Therefore, supplements such as R1,3-butanediol or ketone esters may be more reliable as they can significantly increase blood βHB concentrations with little to no GI distress and risk of salt mineral overdose.

### Challenges and Side-Effects of Ketone Therapies

Ketone therapies are associated with some implementation challenges and side-effects. The foremost challenge with any KD is the requirement for strict compliance to a particular dietary program over an extended period of time, which can for various reasons be impractical or unpalatable for patients. Additionally, a common side-effect of the KD, typically reported by healthy individuals adopting a self-administered KD for weight loss or athletic performance, is “keto flu”, a group of flu-like symptoms including headache, nausea, fatigue, gastrointestinal disruption, and “brain fog” that tend to onset within the 1st week of starting a KD and diminishing around a month later (Bostock et al., 2020). Reportedly mild gastrointestinal side-effects have also been observed in a cohort of healthy participants ingesting exogenous ketone drinks (Stubbs et al., 2019). Exogenous ketones may also produce acute cardiac effects, with ketosis potentially affecting biventricular function, left ventricular and atrial strain, and heart rate (Selvaraj et al., 2022). While small, these studies on gastrointestinal and cardiac effects raise questions surrounding the long-term safety of ketosis in patients with pre-existing gastric or cardiac health problems that would best be addressed in larger-scale future studies.

## Potential of Lactate Therapy for TBIs

Exogenous lactate supplementation has also been considered as a therapeutic option in TBI patients as lactate is known to be utilized by the healthy brain and can support glycemia without inducing a hyperglycemic state since it is hepatically regulated. Various preclinical animal studies are reviewed herein.

Lactate has been shown to have neuroprotective effects similar to ketone supplementation, as reviewed by Patet et al. (2016). Holloway et al. (2007) examined the effects of L-lactate prepared at four different concentrations and administered *via* IV infusion to rats who had received FPIs 11–15 days post-injury. Rats receiving 100 mM of L-lactate were found to have improved MWM test results when compared to saline-treated rat analogs. L-lactate rats also showed a lower reduction in cortical ATP compared to saline-treated animals and no lactic acidosis was observed, indicating the relative safety of the treatment. Sodium L-lactate infusions were studied by Wolahan et al. (2018) in 11 TBI patients who found that net cerebral lactate release was augmented to net cerebral uptake following infusion, with no adverse effects on systemic glucose reported. Hypertonic lactate has also been studied by Bouzat et al. (2014), where patients with severe TBIs were administered with a short infusion. Systemic increases in lactate were accompanied by cerebral increases of lactate and pyruvate, indicating the brain’s ability to draw on systemic lactate supplies, as well as a clinically observable increase in cerebral glucose.

A related role of lactate as a supportive supplement to protect supplies of glucose. This is thought to occur through its action as a signaling molecule where increases in cerebral lactate induce downregulation of glycolysis, facilitating the metabolism of lactate coming from astrocytes into pyruvate. Brooks and Martin (2015) reportedly showed that lactate supplementation *via* a 2 mM lactate clamp did not decrease glucose uptake and raised total cerebral carbohydrate uptake from lactate. In this respect, they postulate that glucose that has been “spared” by the lactate can then be used in other neuroprotective metabolic pathways like the PPP (Bartnik et al., 2005; Dusick et al., 2007; Bouzat et al., 2014). Similarly, Glenn et al. (2015) reported on the “lactate shuttling” role of lactate in dual [6,6-^2^H2]- [3-^13^C] tracer studies where it was found that during periods of suppressed glucose uptake in TBI patients, lactate tracer uptake was comparable to healthy controls indicating that it could be used to compensate for decreased glucose metabolism.

Similar to ketone preparations, the safety of various lactate preparations in humans is an ongoing consideration that needs to be addressed prior to large-scale studies in TBI patients. The safety of short infusions of a sodium lactate preparation has been demonstrated by Miller et al. (2005) who showed manageable sodium and alkalotic stresses in healthy controls and Bouzat et al. (2014) who were able to show uncompromised renal and respiratory function in TBI patients. Other lactate formulations like organic salts (arginyl lactate), and esters (glycerol tri-lactate or N-acetyl lactate) still need to be safely tested in humans.

## TBI Vs. Dementia: A Lesson for Long-Term Care

Speculation surrounding the relationship between TBIs and neurodegenerative processes like dementia has gained greater public interest in the last 5 years due to studies, which are long spanning over 70 years and feature cohorts in excess of 20,000, that have demonstrated a positive correlation between professional football players who are more likely to regularly sustain low-level head injuries and TBIs throughout their career when compared to the average population, and increased risk of death due to neurodegenerative disease (Mackay et al., 2019). The studies in particular identify a form of dementia called chronic traumatic encephalopathy (CTE) that shares signs with Alzheimer’s disease (Vann Jones et al., 2014; Ling et al., 2017). While the acute mechanism of injury and disease progression is clinically very different, there are some long-term compelling metabolic similarities between dementia and TBIs, which raises the question of whether neuroketo therapeutic insight gleaned from TBI cohorts could be utilized in studies of dementia and Alzheimer’s disease (AD).

### Pathological Commonalities

Dementia is a set of neurodegenerative signs and symptoms that can be sub-divided into various more specific conditions based on the apparent mechanism of disease progression. These include AD, Parkinson’s disease, frontotemporal dementia (FTD), Lewy body dementia (LBD), CTE, and amyotrophic lateral sclerosis (ALS). A pathological relationship between TBI and AD in the form of Aβ plaque and tau deposition has been established from post-mortem and imaging studies.

Autopsies of patients below the average age of dementia onset who succumbed to TBI in days to weeks following their injuries showed a diffuse distribution of Aβ plaque reminiscent of that observed in AD (Johnson et al., 2010; Perry et al., 2016). It is uncertain why such TBI patients exhibit such comparatively rapid accumulation of plaque, but it is broadly thought to be associated with vascular and BBB disruption, which has been shown to cause Aβ accumulation and ischemia (Iadecola, 2013; Pluta et al., 2013; de Silva and Faraci, 2016; Wolters et al., 2017). Plasma Aβ42 has indeed been shown to increase during transient hypoxia in healthy subjects (Gren et al., 2016), which would then make Aβ accumulation in TBI patients at a significant risk of extended hypoxia seem reasonable.

Similarly, tau pathology has been observed in both CTE and AD. It is thought in the case of CTE that tauopathy is linked to cerebrovascular damage and dysfunction, as it has been observed specifically around small cortical blood vessels and in the sulci (Omalu et al., 2005; Goldstein et al., 2012; Mckee et al., 2018). Tau accumulation post-TBI could be associated with mechanical stress disrupting microtubule networks leading to diffuse axonal injury, but could also be associated with oxidative stress, which has been shown to affect phosphorylation patterns (Kátai et al., 2016). It would seem then that cerebrovascular damage from TBI could act as an early-stage trigger of AD-like pathology.

### Metabolic Similarities

Disturbance of glucose uptake and metabolism, which has been demonstrated as a key feature in TBIs, has also been characterized as a feature of AD. Even prior to the emergence of clinical symptoms of cognitive impairment, impaired cerebral glucose metabolism has been evidenced as an early warning sign of AD (Mosconi, 2013; Mullins et al., 2018). The first sign is typically decreased capacity for cerebral glucose transport through a reduction in GLUT1 and GLUT3 transporters, which has been evidenced in both human patients and mice models with an overexpression of Aβ (Liu et al., 2004; Hooijmans et al., 2007; Ding et al., 2013). Additionally, the mitochondrial pyruvate dehydrogenase complex (PDC) that plays a role in glucose homeostasis has been found to be decreased in AD along with decreased cytochrome c oxidase activity. The depleted activity of these factors both induce an abnormality in glucose metabolism, which then cyclically contributes to neuronal perturbation. A major similarity then between AD and TBI metabolic dysfunction is the incidence of depressed glucose uptake and glycolysis. It is perhaps the case that an inability to meet the glucose-derived energy demands of the brain in TBI could trigger cascades that elicit AD-like pathologies (Xu et al., 2021). For example, insufficient energy *in vivo* has been shown to trigger phosphorylation of eIF2α, which may promote amyloidogenesis (O’Connor et al., 2008).

### Potential Neuroketotherapeutics for Dementia

Ketogenic therapies have been trialed in a small number of AD patients to address some of the main metabolic features of the disease including impaired glucose metabolism, mitochondrial dysfunction, and amyloid-β (Aβ) plaque accumulation.

In preclinical work, PET studies have shown that AD rats are able to uptake AcAc and BHB in a similar manner to age-matched healthy controls (Castellano et al., 2015). This indicates that KBs may be able to provide the brain with energy substrates that are otherwise unavailable in AD patients with glucose metabolism dysfunction. Further, it appears that such supplementation is associated with reduced pathology and symptoms. For example, exogenous administration of 2-deoxy-D-glucose was associated with reduced AD pathology (Yao et al., 2011), and improved astrocyte metabolism has been shown to reduce memory impairment (Gibbs et al., 2009).

In clinical work, MCT and ketone ester supplements have been shown to raise ketone levels and improve cognition in AD patients (Reger et al., 2004; Newport et al., 2015). Epidemiological studies have also demonstrated a correlation between ketosis and a reduced risk of AD (Reger et al., 2004; Henderson et al., 2009). In both studies, higher plasma ketone was associated with better cognitive function and performance in memory tasks. It was also found that when screening for apolipoprotein-E4 (APO-E4), which is a mutation that confers higher lifetime AD risk, patients testing positive for APO-E4 exhibited a reduced response to exogenous ketone support. Dietary-induced ketosis was studied by Krikorian et al. (2012) who found that the AD patients in the low-carbohydrate KD performed better memory in memory tasks compared to a control, but not significantly better in executive function tasks.

Future work that could be explored includes cases where tau pathologies are caused by oxidative stress as neuroketo therapeutics that have been shown to reduce oxidative stress could be employed, as well as studies exploring whether sustained ketogenesis results in reduced white matter deterioration.

## Conclusion

Following TBI, cerebral blood flow decreases, inducing hypoxia, ischemia, and potential hypermetabolism acutely either in the form of hyperglycolysis or increased glucose uptake to be funneled to the PPP or hypometabolism in months to years after, leading to metabolic dysfunction and increased risk of long-term damage. The increased demand for glucose is complemented by the increased lactate contribution to gluconeogenesis. Lactate also plays a supportive role to protect supplies of glucose. This established the high demand for energy as well as the limited pool of substrates that can bypass the BBB to provide to the brain.

The introduction of commercially available exogenous ketones enabled a research tool to probe the physiological state of ketosis in a reliable and reproducible manner as well as bypassing the restrictions and adherence issues of KD. Early pre-clinical studies showed the great potential of exogenous ketones in mitigating and ameliorating the long-term damage of TBI. However, getting the sample size as well as the right study population is still a challenge for studies in humans. Although the potential for neuroketo therapeutics is very promising (see Table 2), there are still some issues that need to be addressed and investigated. Firstly, is there a threshold of blood ketone concentrations needed to confer benefits to TBI patients and if so, how high should the concentrations be. Secondly, does exogenous ketone confer more benefits in an acute TBI setting when glucose metabolism is increased or in a more chronic setting where there is a clear depression of substrate metabolism. Thirdly, given the increased lactate utilization and unaffected ketone uptake in TBI patients, are there synergistic benefits for lactate and ketone supplementation for TBI as a compensatory alternative substrate to spare the supplies of glucose for PPP and recovery mechanisms, as well as supplying enough ATP for brain function at the same time. In addition to the scientific aspects, there are other factors to be considered as well, such as the different forms of exogenous ketone to be administered, and the difference in cost, taste, and pharmacokinetics as supplementation for chronic TBI patients may require longer-term dosing and the cost, taste, and need for multiple dosing per day may deter adherence. Ultimately, ketones and lactate can be linked as robust “metabolic glucose-alternates” that can drive a majority of brain energy metabolism. This suggests an avenue of exploration in the solution set of metabolic substrates to be assessed for utility as TBI therapeutics.

**Table 2:** Characteristics of brain injury vs. effects of ketones.

Change induced by brain injury	Potential role for ketones
Decreased ATP production	Increased ATP production
Increased oxidative and free radical damage to structural proteins and lipids	Decreased ROS; increased antioxidant capacity
Increased glutamate excitotoxicity	Reduced glutamate synthesis
Increased apoptosis/loss of neurons	Reduced apoptosis; increased BDNF
Possibility for seizures	Anti-seizure effects

The insights on metabolic dysfunction that leads to the mechanism of damage by TBI have allowed researchers to investigate the potential of various interventions and supplementations. The unified metabolic dysfunction displayed across both TBI and dementia suggests a global avenue to investigate and apply any successful interventions and treatments to the at-risk populations for both conditions such as athletes, the military, or the elderly. We hypothesize that supplementation of exogenous ketone in both acute and chronic settings will not only help mitigate the initial damage caused by the injury but by doing so, decrease the risks for longer-term disabilities such as epilepsy, post-traumatic neuropsychiatric symptoms, motor and cognitive deficiencies, and an increased risk of neurodegenerative diseases later in life.

## Author Contributions

NO was responsible for the writing of the manuscript. GW and LM were responsible for editing and writing the manuscript. All authors contributed to the article and approved the submitted version.

## Conflict of Interest

LM is the Research Lead of Health Via Modern Nutrition Inc. (H.V.M.N.), which develops and commercializes products based on ketosis. GW is the founder and Executive Chairman of Health Via Modern Nutrition Inc. (H.V.M.N.). The authors declare that the results of the study are presented clearly, honestly, and without fabrication, falsification, or inappropriate data manipulation. The remaining author declares that the research was conducted in the absence of any commercial or financial relationships that could be construed as a potential conflict of interest.

## Publisher’s Note

All claims expressed in this article are solely those of the authors and do not necessarily represent those of their affiliated organizations, or those of the publisher, the editors and the reviewers. Any product that may be evaluated in this article, or claim that may be made by its manufacturer, is not guaranteed or endorsed by the publisher.
